# Characterizing correlations in partial credit speech recognition scoring with beta-binomial distributions

**DOI:** 10.1121/10.0024633

**Published:** 2024-02-01

**Authors:** Adam K. Bosen

**Affiliations:** Center for Hearing Research, Boys Town National Research Hospital, Omaha, Nebraska 68131, USA adam.bosen@boystown.org

## Abstract

Partial credit scoring for speech recognition tasks can improve measurement precision. However, assessing the magnitude of this improvement with partial credit scoring is challenging because meaningful speech contains contextual cues, which create correlations between the probabilities of correctly identifying each token in a stimulus. Here, beta-binomial distributions were used to estimate recognition accuracy and intraclass correlation for phonemes in words and words in sentences in listeners with cochlear implants (*N* = 20). Estimates demonstrated substantial intraclass correlation in recognition accuracy within stimuli. These correlations were invariant across individuals. Intraclass correlations should be addressed in power analysis of partial credit scoring.

## 1. Introduction

Speech recognition tasks are used in clinical and experimental settings to assess an individual's speech recognition accuracy. For these tasks to be useful, they must collect sufficient data from the individual to provide a precise estimate of their ability. However, this need must be weighed against the cost of collecting the data. This cost is a particular concern in clinical settings, where clinicians have limited time to administer and score such assessments. One approach to improving measurement precision or decreasing the amount of data that need to be collected to achieve a target precision is partial credit scoring, i.e., by scoring a word recognition task by the number of phonemes the participant correctly identified rather than by whether the entire word was correct (Billings *et al.*, 2016). While the precision of measurements obtained with whole item scoring is well-established (Thornton and Raffin, 1978), establishing the precision of partial credit scoring is less straightforward when recognizing each token within a stimulus depends on whether other tokens were recognized. When speech is meaningful, contextual cues from neighboring phonemes in words or words in sentences can support identification (Boothroyd and Nittrouer, 1988), which renders correct identification of any token in a stimulus dependent on whether other contextually related tokens were correctly identified. As a result, the amount of information obtained per stimulus for meaningful stimuli is somewhere between one binary success or failure (i.e., the amount of information obtained *via* whole item scoring) and a number of successes or failures equal to the number of tokens in the stimulus (i.e., recognition accuracy is independent for each token in the stimulus). In terms of measurement precision, the most information is obtained per trial when recognition accuracy is independent across tokens. The least information is obtained per trial when there could be so much contextual information that identifying any one token in the stimulus is sufficient to identify the entire stimulus, which would result in no advantage to partial credit scoring over whole item scoring. In most cases, meaningful speech stimuli fall somewhere in between, with some degree of contextual dependence.

Mathematically, partial credit scoring can be described as sampling from a binomial random process. For a given trial *t* and participant *p*, the participant will correctly identify *X*_tp_ tokens out of a total of *n*_t_ tokens, with the probability of correctly identifying each token determined by the participant's speech recognition accuracy, π_p_,

$$\begin{array}{c} X_{tp}\;∼\;\mathrm{Binomial}(n_{t},\pi_{p}). \end{array}$$ (1)

When the probability of identifying a token is invariant across tokens, recognition accuracy can be modeled as a constant value, *μ*_p_,

$$\begin{array}{c} \pi_{p}=\mu_{p}. \end{array}$$ (2)

However, as described above, the assumption of independence is often violated in meaningful speech. This case can be modeled by randomly sampling *π_p_* from a beta process,

$$\begin{array}{c} \pi_{p}∼\;\mathrm{Beta}\left(\alpha_{p},\beta_{p}\right). \end{array}$$ (3)

Combined, sampling from a beta process to obtain π_p_ and then sampling from a binomial process to obtain X_tp_ yields samples from a beta-binomial process. The beta-binomial distribution is a generalization of the binomial distribution. This generalization allows the beta-binomial distribution to take on a variety of shapes, ranging from being equivalent to the binomial distribution to a U-shaped function in which recognition accuracy is predominantly all-or-nothing. This latter shape is of particular interest here because it describes the case where recognition accuracy for any given token is dependent on recognition accuracy for other tokens within the trial, which would occur when the listener uses contextual information to accurately infer the identity of tokens they did not actually recognize. Formally, when the beta-binomial distribution deviates from the binomial distribution, it is because the variance of the beta-binomial distribution is greater than the variance of the binomial distribution, which is called overdispersion. While multiple parameterizations of the beta-binomial distribution exist, one that is theoretically intuitive for analyzing speech recognition data uses two parameters: *μ* is the mean probability of accurately identifying a token and ρ (alternatively denoted φ in some sources) is the intraclass correlation, which determines the overdispersion of the distribution,

$$\begin{array}{c} \mu_{tp}=\frac{n_{t}\alpha_{p}}{\alpha_{p}+\beta_{p}}, \end{array}$$ (4)

$$\begin{array}{c} \varrho_{p}=\frac{1}{\alpha_{p}+\beta_{p}+1}. \end{array}$$ (5)

**Fig. 1.**
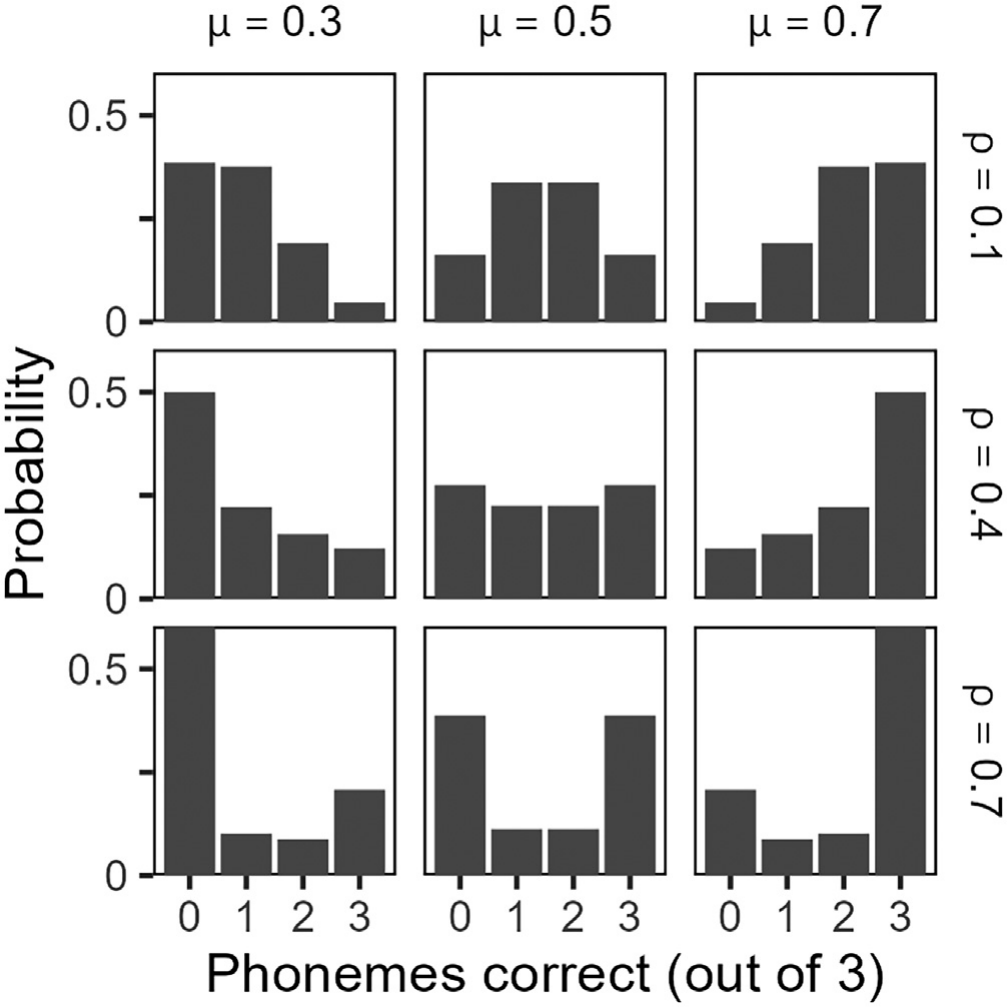
Example beta-binomial distributions of phoneme recognition accuracy within monosyllabic words across varying levels of correct response probability (*μ*) and intraclass correlation (ρ). Nonzero values of ρ indicate overdispersion of recognition accuracy across phonemes within words, which reflects the use of context to facilitate recognition.

Example distributions across varying levels of *μ* and ρ are shown in Fig. 1. At the limits, when ρ approaches zero, the beta-binomial distribution reduces to the binomial distribution, and when ρ approaches one, all tokens within a stimulus are either correctly identified or incorrectly identified. *μ* and ρ can vary orthogonally, such that increasing ρ makes the distribution more U-shaped (i.e., increased variance) without affecting the mean.

In the current work, Bayesian analyses were used to compare binomial and beta-binomial models of speech recognition accuracy. Beta-binomial distributions have been shown to account for overdispersed psychophysical task performance in psychometric function estimation (Fründ *et al.*, 2011; Schütt *et al.*, 2016) and have been used to model speech reception psychometric functions for speech recognition in noise (Hu *et al.*, 2015). The current work extends these previous studies beyond estimating psychometric functions to an examination of individual differences in a constant stimulus speech recognition task. Specifically, models were used to estimate group and individual values of *μ* and ρ for word and sentence recognition data from individuals with cochlear implants. Individuals with cochlear implants dramatically vary from one another in speech recognition accuracy (Gifford *et al.*, 2018), which has been attributed to a wide variety of factors (Holden *et al.*, 2013). Individual differences in the resolution of the auditory signal conveyed by the auditory pathway in response to electrical stimulation and the cognitive ability of the listener to interpret such signals both affect their speech recognition accuracy (O'Neill *et al.*, 2019; Tamati *et al.*, 2020). Cognitive ability plays a role in “top-down” restoration of the degraded signals provided by a cochlear implant (Moberly and Reed, 2019), so I hypothesize that individuals in this population will differ from one another in their use of context, which would manifest as individual differences in intraclass correlation ρ, in addition to known individual differences in the probability of accurate recognition *μ*. Participants in the current study were tested with minimum speech test battery consonant nucleus consonant (CNC) words (MSTB, 2011) because these are linguistically simple stimuli that are commonly used to assess clinical speech recognition outcomes. Participants were also tested with perceptually robust English sentence test open-set (PRESTO) sentences (Gilbert *et al.*, 2013) because these sentences are long and semantically complex. I hypothesize that individual differences in ρ are most likely to be evident in PRESTO sentence recognition because the complexity of these sentences provides the opportunity to use semantic context to fill in missing information. Additionally, if ρ is substantially greater than zero, then the precision of recognition accuracy estimates will decrease more slowly as a function of the number of trials than if ρ is close to zero (Schütt *et al.*, 2016), which would reduce the effective amount of information obtained per trial (Fründ *et al.*, 2011). I simulated the measurement precision of partial credit scoring for CNC words as a function of the number of tested words, which was compared to measurement precision with whole word scoring (Thornton and Raffin, 1978).

## 2. Methods

### 2.1 Participants

Twenty individuals with at least 1 year of cochlear implant use participated in this study (eight males, 12 females, mean age was 60 years with a range of 22–76 years). Participant information is provided in the supplemental Open Science Foundation repository (see Data Availability). All participants provided informed consent and were compensated for their participation. The study was approved by the Boys Town National Research Hospital Institutional Review Board.

### 2.2 Stimuli and procedure

Participants listened to and repeated MSTB CNC words and PRESTO sentences that were presented from a loudspeaker in a sound-treated booth at a mean level of 65 dB SPL. All participants used their everyday cochlear implant processor set to their default clinical program. Participants who had residual acoustic hearing removed their hearing aids and wore an ear plug to render stimuli inaudible to the ear with residual hearing. Verbal responses were recorded for offline transcription and scoring.

A total of 250 CNC words (MSTB lists 1, 2, 4, 5, and 10) and 36 PRESTO sentences (lists 13 and 17; 152 keywords total) were presented to each participant in a fixed order. Prior to testing with each stimulus set, three practice MSTB words (“duck,” “bomb,” and “June”) and three sentences from PRESTO List 1 were presented to familiarize the participant with the stimuli. Participants were instructed to press a button on a computer mouse to initiate playback of each stimulus, then listen to the stimulus and repeat aloud what they thought was said. The task was self-paced, and breaks were offered between lists.

### 2.3 Data analysis

CNC word responses were transcribed in Klattese. Edit distance was used to calculate the number of edits required to transform the response to the target word and the number of phonemes correct was calculated as three minus the number of edits, to a minimum of zero phonemes correct (Gonthier, 2022). PRESTO sentences were scored by the number of keywords correct using scoring criteria provided with those sentences. PRESTO sentence recognition data were previously reported by Bosen *et al.* (2021).

Four models were used to estimate individual differences in model parameters *μ* and ρ for both stimulus sets. The first model assumed that the number of correct responses *X*_tp_ in each trial arose from a binomial distribution, with mean accuracy *μ*_p_ varying across individuals but constant across trials,

$$\begin{array}{c} X_{tp}\;∼\;\mathrm{Binomial}(n_{t},\mu_{p}). \end{array}$$ (6)

The second model assumed that the number of correct responses in each trial arose from a beta-binomial distribution. Recognition accuracy for each trial, π_tp_, was sampled from a beta distribution with mean accuracy and intraclass correlation *μ*_p_ and ρ_p_ varying across individuals,

$$\begin{array}{c} X_{tp}\;∼\;\mathrm{BetaBinomial}(n_{t},\mu_{p},\varrho_{p}). \end{array}$$ (7)

The third model also assumed a beta-binomial distribution, but fixed ρ_group_ to a constant value across all participants. Comparing the second and third model determined whether individual variability in intraclass correlation ρ_p_ provides a better explanation of the data than fixed intraclass correlation ρ_group_,

$$\begin{array}{c} X_{tp}\;∼\;\mathrm{BetaBinomial}(n_{t},\mu_{p},\varrho_{\text{group}}). \end{array}$$ (8)

**Fig. 2.**
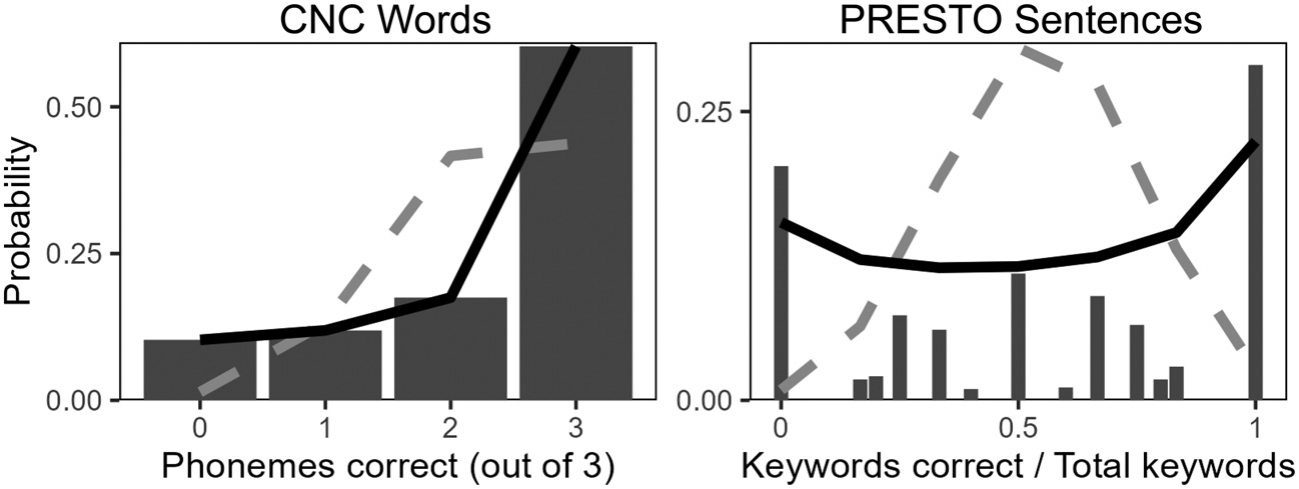
Group-level distribution of correct phonemes within MSTB CNC words and words within PRESTO sentences are shown as bars. Beta-binomial distributions are shown as solid black lines and binomial distributions are shown as dashed gray lines. PRESTO sentences have a variable number of keywords per trial, so number of keywords correct is shown as a proportion out of total keywords.

Finally, the fourth model fixed *μ*_group_ and ρ_group_ to constant values across all participants. This model was used to examine the group-level distribution of the data (see Fig. 2 below) but was not compared to the other models,

$$\begin{array}{c} X_{t}\;∼\;\mathrm{BetaBinomial}(n_{t},\mu_{\text{group}},\varrho_{\text{group}}). \end{array}$$ (9)

Beta-binomial model parameters were estimated in two ways. First, the VGAM package (v1.1.8; Yee, 2010) in R (v4.2.0; R Core Team, 2022) was used to estimate group- and individual-level parameter values for each stimulus set as an easy-to-use method of obtaining descriptive statistics to show group trends and as a reference for developing more complex Bayesian models. Second, the Stan programming language (v2.26.1; Carpenter *et al.*, 2017), using the RStan interface (v2.26.11; Stan Development Team, 2020) was used to compare the three models of task performance described above. Priors for *μ* and ρ were set to Beta(2,2) to provide a weakly informative but nonuniform prior, although a prior sensitivity analysis indicated that the choice of prior did not substantially affect model parameter estimates (see Data Availability for information about the supplemental OSF repository). Models were compared by using Pareto smoothed importance-sampling leave-one-out cross-validation (Vehtari *et al.*, 2017) to estimate expected log pointwise predictive density (ELPD), which quantifies goodness of fit. If two models have a difference in ELPD of greater than four and the standard error of the difference is smaller than the difference, then the model with the higher ELPD is a better explanation for the data (Sivula *et al.*, 2020). For an overview of the workflow when using Bayesian modeling and an example of its use in auditory research (see Gelman *et al.*, 2020; McMillan and Cannon, 2019).

To estimate measurement precision for mean accuracy using phoneme-level scoring in MSTB word lists when intraclass correlations are present, 10 000 draws from two distributions were made across a range of 10–100 words tested. Conditions simulated were whole word scoring,

$$\begin{array}{c} X\;∼\;\mathrm{Binomial}\left(n=1,\;\mu =0.5\right) \end{array}$$ (10)

and phoneme scoring using the most likey value for ρ_group_ (see results),

$$\begin{array}{c} X\;∼\;\mathrm{BetaBinomial}\left(n=3,\;\mu =0.5,\;\varrho =0.35\right). \end{array}$$ (11)

The standard deviation of mean accuracy across draws for each number of words was used as a metric of measurement precision. For this simulation, a fixed recognition accuracy of *μ* = 0.5 was used because the standard deviation of the mean is greatest at this value for a binomial distribution (Thornton and Raffin, 1978). I then calculated the minimum number of words required to equate the standard deviation of the mean for phoneme scoring with the standard deviation for whole word scoring of responses to 50 words, because 50 word lists are often used in clinical assessment (MSTB, 2011).

## 3. Results

Figure 2 shows the group-level distribution of phonemes correct in CNC words and words correct in PRESTO sentences. For CNC words, participants correctly identified an average of 60% of CNC words and *μ*_group_ = 76% of phonemes within those words. For PRESTO sentences, participants correctly identified an average of *μ*_group_ = 55% of keywords. Binomial distributions using these values of *μ*_group_, shown as dashed gray lines, generally fail to capture trends in phonemes or keywords correct. For the CNC words, the binomial distribution overestimates the probability of correctly identifying two out of three phonemes and underestimates the probability of correctly identifying all three keywords. In the majority of PRESTO sentences, participants either correctly identified every keyword or none of them (proportions of one or zero, respectively), although the binomial distribution predicts that either outcome should be unlikely. For beta-binomial distributions, most likely values of intraclass correlations were ρ_group_ = 0.46 for CNC words and ρ_group_ = 0.42 for PRESTO sentences. Beta-binomial distributions using these values, shown as solid black lines, are capable of representing these trends. This observation indicates that substantial intraclass correlations exist in responses to these stimuli, as expected.

**Fig. 3.**
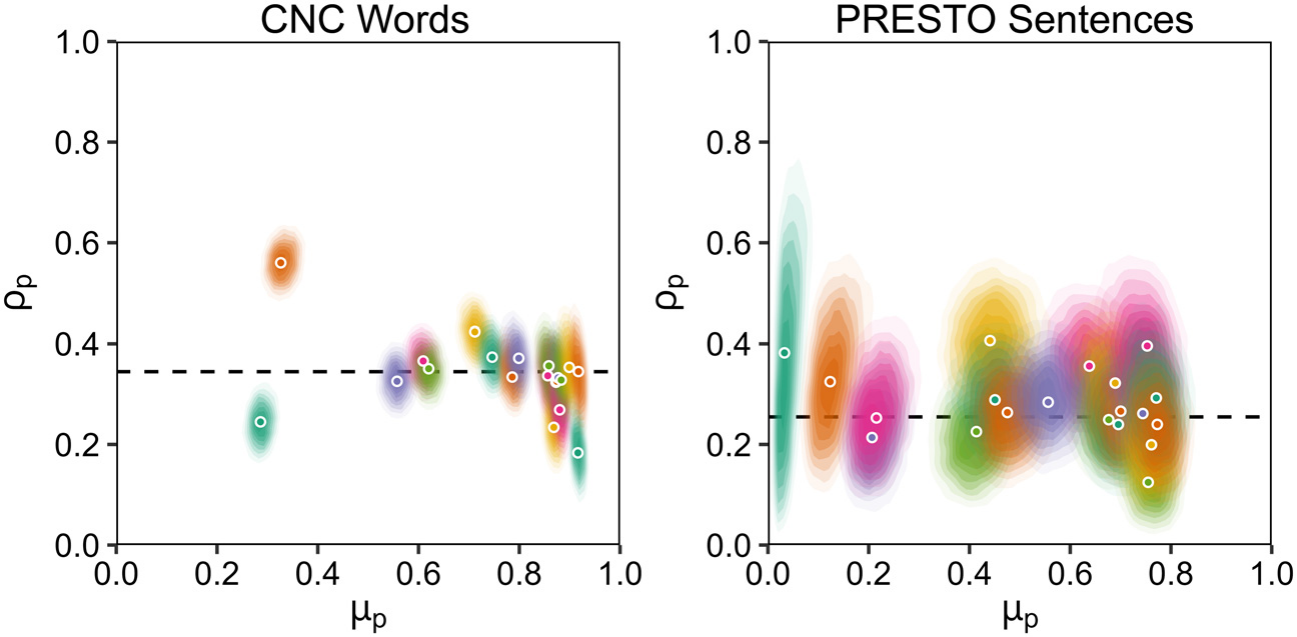
Individual differences in recognition accuracy *μ*_p_ and intraclass correlation ρ_p_ estimated *via* Bayesian modeling. Most likely values for model parameters for each participant are shown as circles, with colors representing different participants. Shaded gradients around each most likely value show the corresponding posterior probability density for both model parameters for that participant, with darker shades corresponding to higher probability density. The dashed black line shows the most likely value for ρ_group_.

Figure 3 shows individual differences in the most likely values of *μ*_p_ and ρ_p_ for CNC words and PRESTO sentences, along with estimated posterior probability densities for each model parameter. Individual data and model fits are provided in the supplemental OSF repository. As expected, individuals substantially vary in recognition accuracy *μ*_p_, with a range of 29%–92% phonemes correct for CNC words and 3%–78% keywords correct for PRESTO sentences. Contrary to my hypothesis, there appears to be little variation in intraclass correlation ρ_p_ across individuals. Comparisons of model fits, which either varied or fixed ρ across participants, shown in Table 1, confirm this observation. For both datasets, fixing the value of ρ_group_ across participants did not yield a substantially worse model fit for CNC words and improved the model fit for PRESTO sentences. Group intraclass correlations were ρ_group_ = 0.35 for CNC words and ρ_group_ = 0.25 for PRESTO sentences. These values of ρ_group_ are lower than when *μ*_group_ is fixed across participants, indicating that individual differences in accuracy *μ*_p_ affect the shape of the group-level distributions shown in Fig. 2. Binomial distributions had a substantially worse fit to the data than either beta-binomial model, confirming that including intraclass correlations is necessary to explain the distribution of recognition accuracy for phonemes in words or keywords in sentences.

**TABLE 1.** Comparison of binomial and beta-binomial models of CNC word and PRESTO sentence data. Expected log pointwise predictive density (ELPD_loo_), number of effective model parameters (p_loo_), and the standard errors for both estimates (SE) were estimated for both model fits using the LOO package in R (Vehtari *et al.*, 2017). Model fits were compared to obtain an estimated difference in expected log posterior density relative to the best-fitting model (ΔELPD) and standard error for the difference.

CNC words						
Model	ELPD_loo_	ELPD_loo_ SE	p_loo_	P_loo_ SE	ΔELPD	ΔELPD SE
$X_{tp}\;∼\;\mathrm{BetaBinomial}(n_{t},\mu_{p},\varrho_{p})$	−4811.8	57.3	38.1	1.0	—	—
$X_{tp}\;∼\;\mathrm{BetaBinomial}(n_{t},\mu_{p},\varrho_{\text{group}})$	−4817.1	56.9	20.6	0.4	−5.3	6.2
$X_{tp}\;∼\;\mathrm{Binomial}(n_{t},\mu_{p})$	−5410.9	74.6	32.9	0.9	−599.1	39.3

PRESTO sentences						
Model	ELPD_loo_	ELPD_loo_ SE	p_loo_	P_loo_ SE	ΔELPD	ΔELPD SE
$X_{tp}\;∼\;\mathrm{BetaBinomial}(n_{t},\mu_{p},\varrho_{\text{group}})$	−990.5	17.9	19.7	0.8	—	—
$X_{tp}\;∼\;\mathrm{BetaBinomial}(n_{t},\mu_{p},\varrho_{p})$	−1002.0	18.3	33.2	1.6	−11.5	2.9
$X_{tp}\;∼\;\mathrm{Binomial}(n_{t},\mu_{p})$	−1090.5	27.1	33.4	1.7	−99.9	16.2

**Fig. 4.**
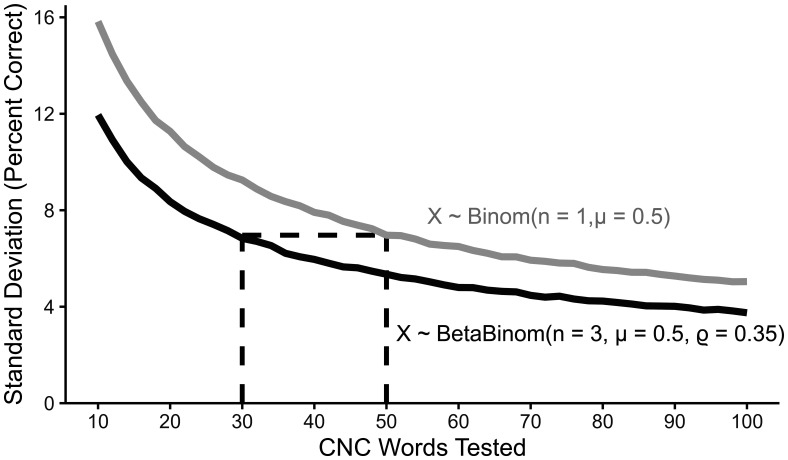
The standard deviation of the mean for repeated draws from whole word scoring (*n* =1, *μ* = 0.5) and a three-phoneme beta-binomial distribution (*n* = 3, *μ* = 0.5, ρ = 0.35) as a function of the number of words tested. Dashed lines show the comparison of the number of trials required to equate standard deviation of the mean for the beta-binomial distribution with whole word scoring for 50 words.

Figure 4 shows the standard deviation of the mean when sampling from a beta-binomial distribution with ρ_group_ = 0.35, which was the value obtained for CNC words. As shown, a minimum of 30 words are required to equate the standard deviation of the mean to a 50-word sample with whole word scoring.

## 4. Discussion

Beta-binomial distributions quantify intraclass correlations when multiple binary outcomes are measured within a sample, such as when scoring the proportion of correct phonemes in words or correct words in sentences. Figure 2 shows that context effects create substantial intraclass correlations that alter the distribution of the number of tokens in each stimulus that are correctly identified. While methods of quantifying the amount of context in a stimulus set have already been established (Boothroyd and Nittrouer, 1988), the use of beta-binomial model fits proposed here provides additional flexibility to incorporate individual differences in recognition accuracy and/or intraclass correlation and compare models that fix or vary these parameters across individuals. My present findings, alongside previous work examining the use of beta-binomial distributions as a model of speech recognition in varying levels of noise (Hu *et al.*, 2015), indicate that recognition of meaningful speech is likely to generally follow this distribution. More broadly, the use of Bayesian statistics enables the estimation of the range of likely values for model parameters (Morey *et al.*, 2016) to characterize individual differences in those parameters, provides additional diagnostic information for identifying models that are good or poor explanations for the data (Vehtari *et al.*, 2017), and provides the flexibility to attempt using any model that can be expressed as a set of statistical formulae.

The practical advantage of using beta-binomial distributions to analyze speech recognition data is that can be used to quantify the benefit of partial credit scoring, as shown in Fig. 4. Here, recognition accuracy for MSTB CNC words in listeners with cochlear implants was examined because this is a common clinical assessment of speech recognition outcomes in a heterogeneous clinical population. Scoring responses by the number of phonemes correct within a word improved measurement precision, but because the intraclass correlation was nonzero, this improvement fell short of what would be expected if recognition accuracy was independent for each phoneme within a word. If phoneme recognition accuracy was independent, only 17 words (50 words/three phonemes) would be required to achieve an equivalent precision to whole word scoring, but with the average intraclass correlation estimated for the CNC words, a total of 30 words is needed. Thus, power analyses, which estimate the amount of data required per participant, need to be designed to account for intraclass correlations to ensure that measures are not underpowered. The tools used to fit beta-binomial distributions in the present work are free and publicly available and example source code is provided in the supplemental OSF repository, which should facilitate adoption of this type of analysis.

Against the expected hypothesis, allowing intraclass correlations to vary across participants in this study did not provide a better explanation for the data than fixing the value of intraclass correlation across participants, as shown in Fig. 3 and Table 1. There were a few individuals for whom the estimated probability density of intraclass correlation for CNC words was unlikely to include the fixed group value, but the addition of individual intraclass correlation model parameters was not necessary to explain most participants' recognition accuracy. This finding could reflect the fact that we recruited only post-lingually deafened adults with no known cognitive impairments. Children who use cochlear implants differ from post-lingually deafened adults in the auditory cues they use to recognize speech (Gifford *et al.*, 2018) and often have heterogeneous delays in language development (Niparko *et al.*, 2010); thus, it is possible that individual differences in intraclass correlation would be more evident in children (see also Nittrouer and Boothroyd, 1990 for evidence in favor of this possiblity in children with normal hearing). Additionally, any cognitive impairment that affects lexical access would likely also alter contextual use. Attentional control and hearing status also interact to determine the effect of masking speech or noise on recognition of target speech (Shinn-Cunningham and Best, 2008), so individual differences in intraclass correlations could be revealed by adding maskers that would produce more all-or-nothing responses (Boothroyd and Nittrouer, 1988). Examining the cases in which intraclass correlations do or do not vary across individuals could inform theories of stream segregation and speech recognition.

A larger sample of clinical data could potentially be used to establish normative values for intraclass correlations in clinical assessments and thereby develop criteria to flag outliers as potential indicators of cognitive or linguistic issues. No additional clinical testing time would be needed to use these criteria, although scoring response by phonemes correct within words does require both more work than whole word scoring and consistency across testers to be meaningful (Billings *et al.*, 2016). To ensure clinical test–retest differences are reliable, it may also be necessary to test for variability in intraclass correlations across test lists, in addition to the previously reported variability in recognition accuracy across MSTB CNC lists (Bierer *et al.*, 2016). The phonemes within words may also affect intraclass correlations because phonemes that tend to be hard to identify with a cochlear implant (Munson *et al.*, 2003) may be inferred from context more often than phonemes that are easier to identify. While the data reported in the present study is insufficiently powered to address these questions, it establishes proof of concept for using beta-binomial distributions as a tool that could be used with extant datasets to provide answers.

## Data Availability

The data and analysis that support the findings of this study are openly available in the Open Science Foundation at https://osf.io/y8t5k/.
